# TRIM31 promotes acute myeloid leukemia progression and sensitivity to daunorubicin through the Wnt/β-catenin signaling

**DOI:** 10.1042/BSR20194334

**Published:** 2020-04-15

**Authors:** Yi Xiao, Taoran Deng, Xi Ming, Jinhuang Xu

**Affiliations:** Department of Hematology, Tongji Hospital, Tongji Medical College, Huazhong University of Science and Technology, Wuhan 430030, China

**Keywords:** acute myeloid leukemia (AML), TRIM31, drug resistance, daunorubicin, Wnt/β-catenin pathway

## Abstract

Tripartite motif (TRIM) 31 is a member of TRIM family and exerts oncogenic role in the progression and drug resistance of several cancers. However, little is known about the relevance of TRIM31 in acute myeloid leukemia (AML). Herein, we investigated the role of TRIM31 in AML. We examined the expression levels of TRIM31 in the blood samples from 34 patients with AML and 34 healthy volunteers using qRT-PCR. The mRNA levels of TRIM31 in human bone marrow stromal cells (HS-5) and five AML cell lines were also detected. Loss/gain-of-function assays were performed to assess the role of TRIM31 in AML cells proliferation, apoptosis and sensitivity to daunorubicin. The expression levels of pro-caspase 3, cleaved caspase 3, Wnt3a, β-catenin, cyclin D1 and c-Myc were measured using Western blot. TRIM31 expression levels were significantly up-regulated in AML patients and cell lines. Knockdown of TRIM31 suppressed cell proliferation and promoted apoptosis in AML-5 and U937 cells. The IC_50_ of daunorubicin was significantly decreased in TRIM31 siRNA (si-TRIM31) transfected cells. Oppositely, induced cell proliferation and decreased cell apoptosis were observed in pcDNA-3.1-TRIM31 transfected cells. Furthermore, knockdown of TRIM31 suppressed the activation of Wnt/β-catenin pathway in AML cells. Activation of Wnt/β-catenin pathway by LiCl abolished the effects of si-TRIM31 on cell proliferation, apoptosis and sensitivity to daunorubicin in AML cells. In conclusion, the results indicated that TRIM31 promoted leukemogenesis and chemoresistance to daunorubicin in AML. The oncogenic role of TRIM31 in AML was mediated by the Wnt/β-catenin pathway. Thus, TRIM31 might serve as a therapeutic target for the AML treatment.

## Introduction

Acute myeloid leukemia (AML) is a kind of leukemia that starts in the myeloid cells, which have the capacities of self-renewing, sustaining malignant populations and producing subclones [1]. AML is the most common acute leukemia and can be very fatal in a short period without suitable intervention. Early and common symptoms of AML include fatigue, increased risk of infection, arthralgia and hemorrhage [2]. Available reports point to the risk factors implicated in the development of AML, such as chemical exposures, ionizing radiation, blood disorders and heredity [3]. In the past decades, the incidence of AML is gradually increasing.

Currently, the management of AML is divided into induction phase and consolidation phase [4,5]. Chemotherapy is a standard treatment option applied in both induction and consolidation phases to destroy leukemic cells [6]. However, this therapy usually presents high toxicity and high risk of recurrence due to the chemoresistance of AML stem cells [4]. Considering these alarming challenges, it is imperative to develop appropriate intervention for AML progression and chemoresistance.

Tripartite motif (TRIM) 31, a member of the TRIM protein family, was found to exert oncogenic role in the development and progression of several tumors. Overexpression of TRIM31 is observed in high-grade glioma tissues and associated with short survival time. Overexpressing TRIM31 promotes the proliferation, invasion and migration of glioma cells [7]. TRIM31 expression is significantly up-regulated in hepatocellular carcinoma (HCC) tissues and significantly correlated with advanced disease status. TRIM31 promotes the malignant behaviors of HCC cells through regulation of mammalian target of rapamycin complex1 (mTORC1) pathway [8]. In addition, TRIM31 has been proven to be associated with radiosensitivity and chemosensitivity in cancers. Knockdown of TRIM31 enhances radiosensitivity of colorectal cancer cells by inducing DNA damage and cell apoptosis [9]. High expression of TRIM31 in pancreatic cancer patients is associated with aggressive phenotype and poor prognosis. TRIM31 overexpression confers gemcitabine resistance in pancreatic cancer cells [10].

Considering the oncogenic roles of TRIM31 in these types of cancers, we speculated that TRIM31 might exhibit similar biological functions in AML. Thus, the aim of the present study was to uncover the role of TRIM31in AML.

## Materials and methods

### Patient samples collection

The study was approved by the Ethics Committee of Tongji Hospital, Tongji Medical College, Huazhong University of Science and Technology (Wuhan, China). A total of 34 patients diagnosed with AML and 34 healthy volunteers were enrolled. Prior informed consents were obtained from all participants. Up to 5 ml whole blood sample was obtained from each participant and used for the detection of TRIM31.

### Cell culture and transfection

Human bone marrow stromal cells (HS-5) and four AML cell lines including AML-5, U937, K-562 and THP-1 cells were obtained from the American Type Culture Collection (ATCC, Manassas, VA, U.S.A.). The cells were cultured in RPMI-1640 or DMEM (Life Technologies, Carlsbad, CA, USA). The medium was supplemented with 10% FBS (Life Technologies).

The TRIM31 siRNA (si-TRIM31), non-targeting scrambled siRNA (si-NC) and TRIM31-overexpressing plasmid (pcDNA-3.1-TRIM31) were procured from GenePharma (Shanghai, China). Cell transfections were carried out using Lipofectamine 2000 (Invitrogen, Carlsbad, CA, U.S.A.) based on the manufacturer’s instructions.

### qRT-PCR

Total RNA was isolated from blood samples using RNAprep Pure Blood Kit (Tiangen Biotech, Beijing, China). Total RNA was extracted from the cell lines using a Total RNA Purification kit (Invitrogen) in accordance with the manufacturer’s protocols. After the assessment of quantity and concentration of RNA, total RNA was used for the reverse transcription reaction with the Prime-Script RT reagent kit (TaKaRa, Dalian, China). Quantitative RT-PCR was performed using an miScript SYBR green PCR kit (Qiagen, Hilden, Germany). The relative mRNA level of TRIM31 was calculated using the comparative 2^−ΔΔ*C*_t_^ method.

### Western blot

Total proteins were extracted from AML-5 and U937 cells using radioimmunoprecipitation assay (RIPA) lysis buffer (Sangon Biotech, Shanghai, China). The protein samples were loaded for electrophoresis and then subjected to Western blot analysis for the measurement of pro-caspase 3, cleaved caspase 3, Wnt3a, β-catenin, cyclin D1 and c-Myc expression levels. The specific primary and corresponding secondary antibodies were obtained from Santa Cruz Biotechnology (Santa Cruz, CA, U.S.A.). The protein bands were detected by enhanced chemiluminescence (ECL) reagent (Thermo Fisher Scientific, Waltham, MA, U.S.A.) and analyzed by ImageJ Software (NIH, Bethesda, MD, U.S.A.).

### MTT assay

MTT assay was performed to assess cell proliferation and sensitivity to daunorubicin. For the cell proliferation assay, AML-5 and U937 cells (1 × 10^4^ cells/well) were plated in a 96-well plate and cultured for adhesion. After incubating for 0, 24, 48, 72, 96 h, each well was added with 20 µl MTT (5 mg/ml, Sigma–Aldrich, St. Louis, MO, U.S.A.) and incubated for additional 4 h. Then, 150 μl DMSO was added the cells to dissolve formazan. Finally, the optical density (OD) at 490 nm was measured using a Microplate Reader (Molecular Devices, Sunnyvale, CA, U.S.A.).

For the chemosensitivity assay, AML-5 and U937 cells (1 × 10^4^ cells/well) were incubated with a series of concentrations of daunorubicin (0, 2, 4, 8, 16 μM) for 24 h. Then the cell viability was measured using MTT assay as described above.

### Cell apoptosis assay

Cell apoptosis was detected by flow cytometry using AnnexinV-FITC/Propidium Iodide (PI) staining kit (Invitrogen). In brief, after transfection, cells were harvested and resuspended in binding buffer. Subsequently, AnnexinV-FITC and PI were added and stained for 15 min at room temperature in dark. Finally, cells were subjected to flow cytometry (BD Biosciences, San Jose, CA, U.S.A.).

### Caspase-3 activity

Caspase-3 activity of AML-5 and U937 cells was measured using a caspase-3 activity assay kit (Nanjing Jiancheng Bioengineering Institute). According to the instructions, cell lysates were incubated with 50 μl Ac-DEVD-pNA for 4 h at 37°C. The absorbance at 450 nm was measured using a microplate Reader (Bio-Rad).

### Statistical analysis

All statistical analyses were performed using Student GraphPad Prism 5 (GraphPad Software, Inc., La Jolla, CA, U.S.A.). The Student’s *t* test or one-way ANOVA was respectively used to assess the significant differences between two groups or among multiple groups. Differences were considered statistically significant when *P*-value <0.05.

## Results

### TRIM31 was highly expressed in AML patients and cell lines

**Figure 1:**
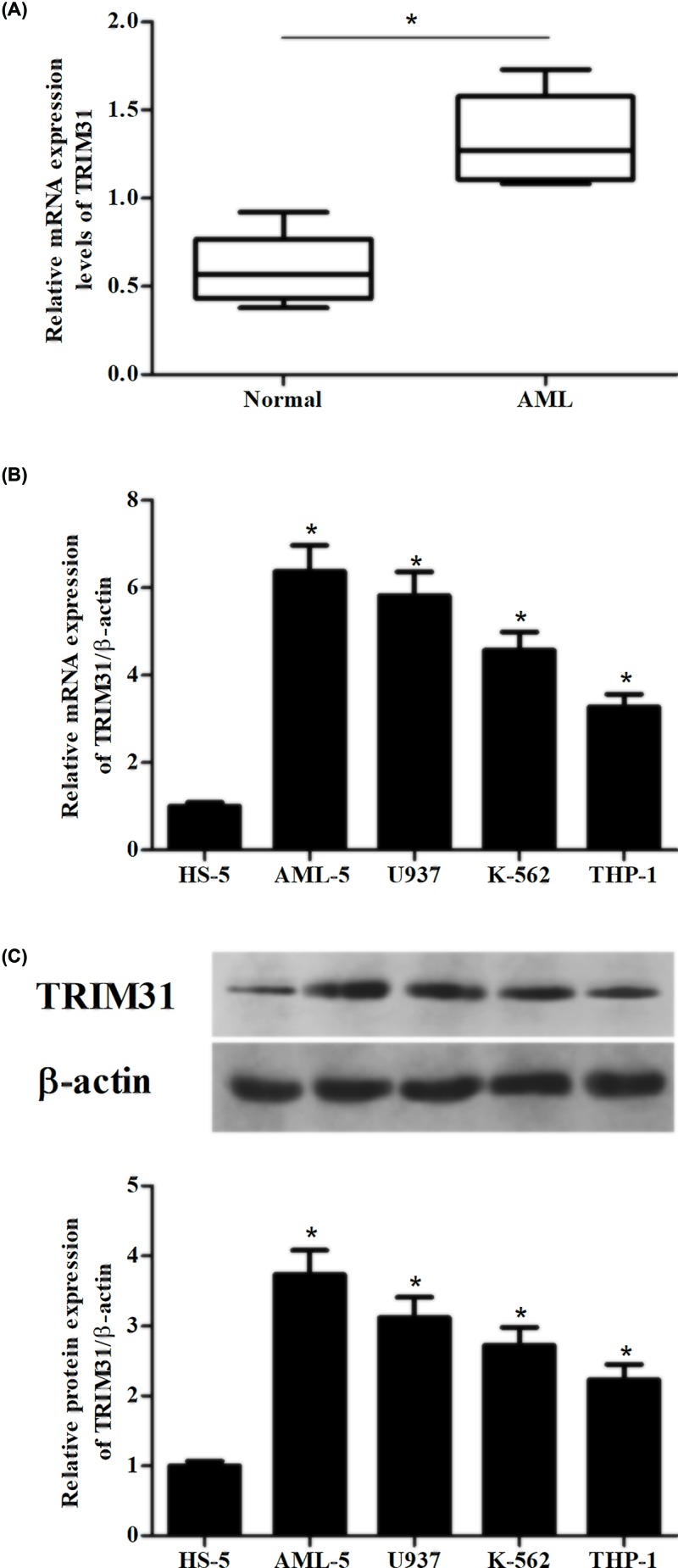
Relative expression levels of TRIM31 in blood samples and cell lines (**A**) Expression levels of TRIM31 in blood samples from AML patients (*n*=34) and healthy volunteers (*n*=34). *P<0.05 compared with control group (healthy volunteers). (**B,C**) The mRNA and protein levels of TRIM31 in human bone marrow stromal cells (HS-5) and four AML cell lines (AML-5, U937, K-562 and THP-1 cells). *P<0.05 compared with HS-5 cells.

In the present study, we first validated the relative expression of TRIM31 in AML patients. Results in Figure 1A showed that up-regulation of TRIM31 expression was observed in blood samples from AML patients compared with samples from healthy volunteers. Next, the TRIM31 expression levels in human bone marrow stromal cells (HS-5) and four AML cell lines (AML-5, U937, K-562 and THP-1 cells) were also detected using qRT-PCR and Western blot. The results showed that highexpression levels of TRIM31 were observed in AML cell lines, especially in AML-5 and U937 cells (Figure 1B,C).

### Knockdown of TRIM31 inhibited the proliferation and induced the apoptosis of AML cells

**Figure 2:**
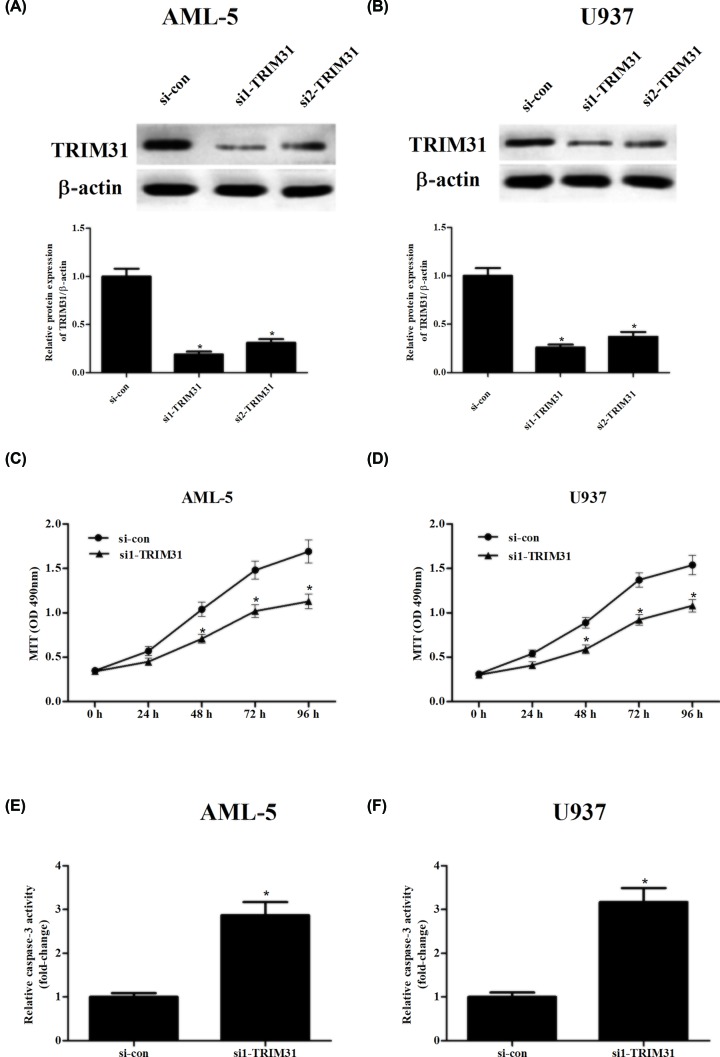
Loss-of-function assays were performed after transfection with si-TRIM31 (**A,B**) AML-5 and U937 cells were transfected with si1/2-TRIM31 or si-con, and transfection efficiency was confirmed by Western blot. (**C,D**) MTT assay was performed to detect AML-5 and U937 cells proliferation. (**E,F**) Caspase-3 activity was carried out to analyze apoptosis of AML-5 and U937 cells. *P<0.05.

Loss-of-function assays were then performed in AML-5 and U937 cells through transfection with si-TRIM31 or si-NC (Figure 2A,B). MTT assay demonstrated that TRIM31 knockdown significantly decreased AML-5 and U937 cells proliferation, compared with si-NC transfected cells (Figure 2C,D). Furthermore, we found that knockdown of TRIM31 significantly promoted caspase-3 activity in AML-5 and U937 cells, respectively, as compared with the si-con group (Figure 2E,F). Similarly, we also found that knockdown of TRIM31 greatly promoted cell apoptosis, increased cleaved caspase 3 expression and reduced pro-caspase 3 expression in AML-5 and U937 cells, respectively (Supplementary Figures S1A,B and S2A,B).

### Overexpression of TRIM31 promoted the proliferation and inhibited the apoptosis of AML cells

**Figure 3:**
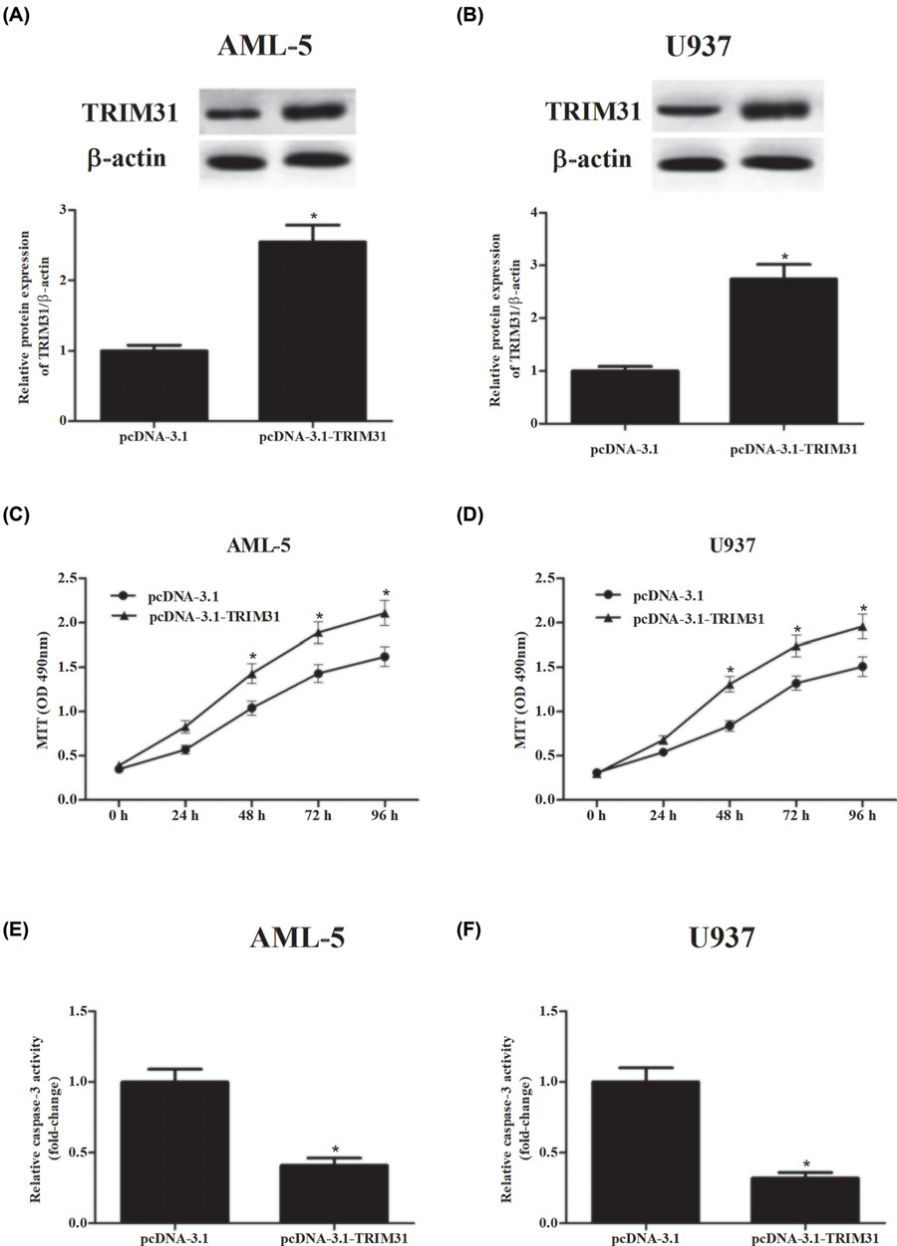
Gain-of-function assays were performed after transfection with pcDNA-3.1-TRIM31 (**A,B**) Western blot was applied to assess the protein levels of TRIM31 after transfection with pcDNA-3.1-TRIM31 or pcDNA-3.1 empty plasmid in AML-5 and U937 cells. (**C,D**) MTT assay was performed to detect AML-5 and U937 cells proliferation. (**E,F**) Caspase-3 activity was carried out to analyze apoptosis of AML-5 and U937 cells. *P<0.05.

Subsequently, gain-of-function assays were performed through transfection with TRIM31-overexpressing plasmid (pcDNA-3.1-TRIM31) or pcDNA-3.1 empty plasmid (Figure 3A,B). As illustrated in Figure 3C,D, after transfection with pcDNA-3.1-TRIM31, AML-5 and U937 cells proliferation were obviously promoted. Meanwhile, pcDNA-3.1-TRIM31 transfection led to significant reduction in caspase-3 activity in both AML-5 and U937 cells (Figure 3E,F). Furthermore, TRIM31 overexpression significantly inhibited cell apoptosis, down-regulated cleaved caspase 3 expression and up-regulated pro-caspase 3 expression in AML-5 and U937 cells, respectively (Supplementary Figures S1C,D and S2C,D).

### Knockdown of TRIM31 enhanced cell sensitivity to daunorubicin

**Figure 4:**
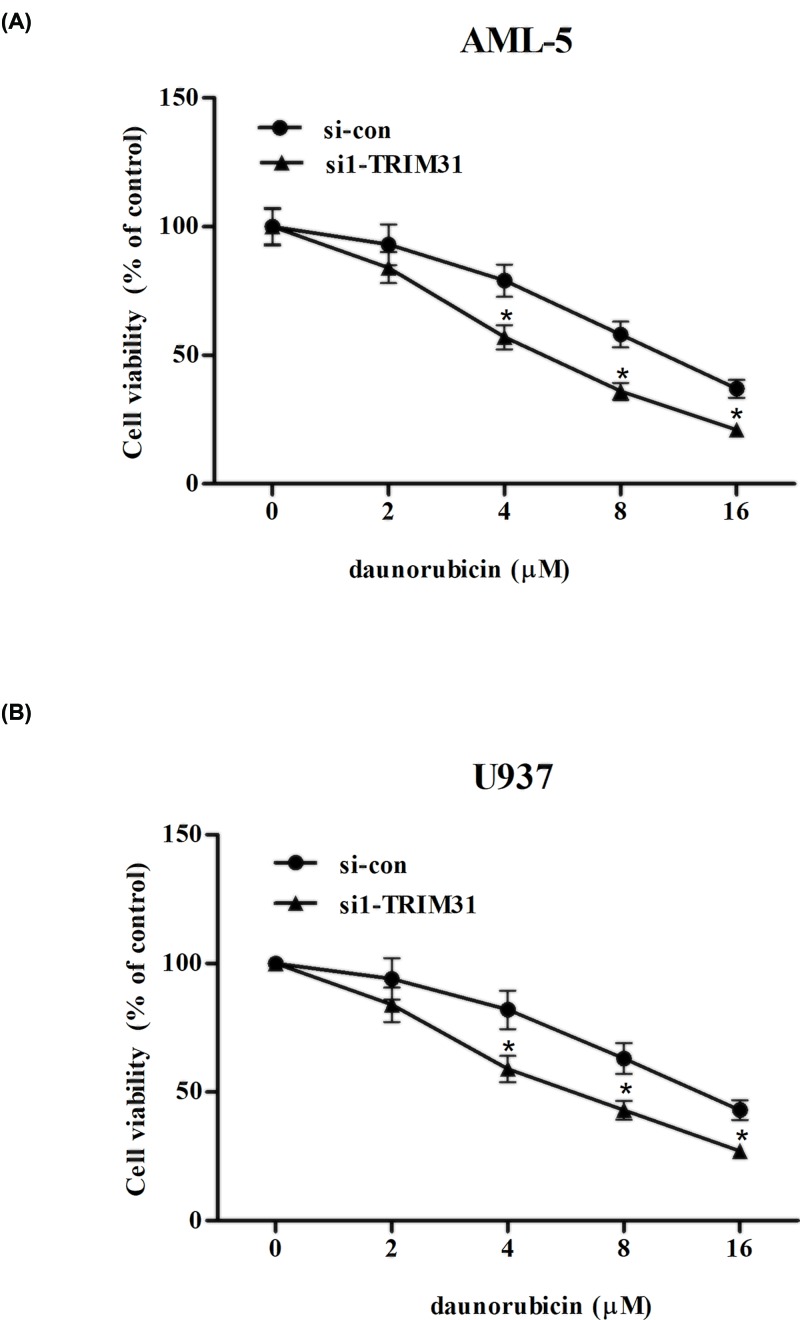
Evaluation of cell sensitivity to daunorubicin after transfection with si-TRIM31 AML-5 and U937 cells transfected with si-con or si1-TRIM31 were treated with various concentrations of ADR (0, 2, 4, 8, 16 μM) for 24 h. Then, cell viability was measured in AML-5 (**A**) and U937 cells (**B**) using MTT assay. *P<0.05.

As shown in Figure 4A,B, daunorubicin significantly reduced cell viability in both AML-5 and U937 cells in dose-dependent manner. The growth rate in si-TRIM31 transfected AML-5 cells was less than that in normal AML-5 cells. Similarly, the growth rate was lower in si-TRIM31 transfected U937 cells than normal U937 cells. The results indicated that daunorubicin sensitivity was significantly increased in si-TRIM31 transfected AML cells.

### Knockdown of TRIM31 suppressed the activation of Wnt/β-catenin signaling pathway in AML cells

**Figure 5:**
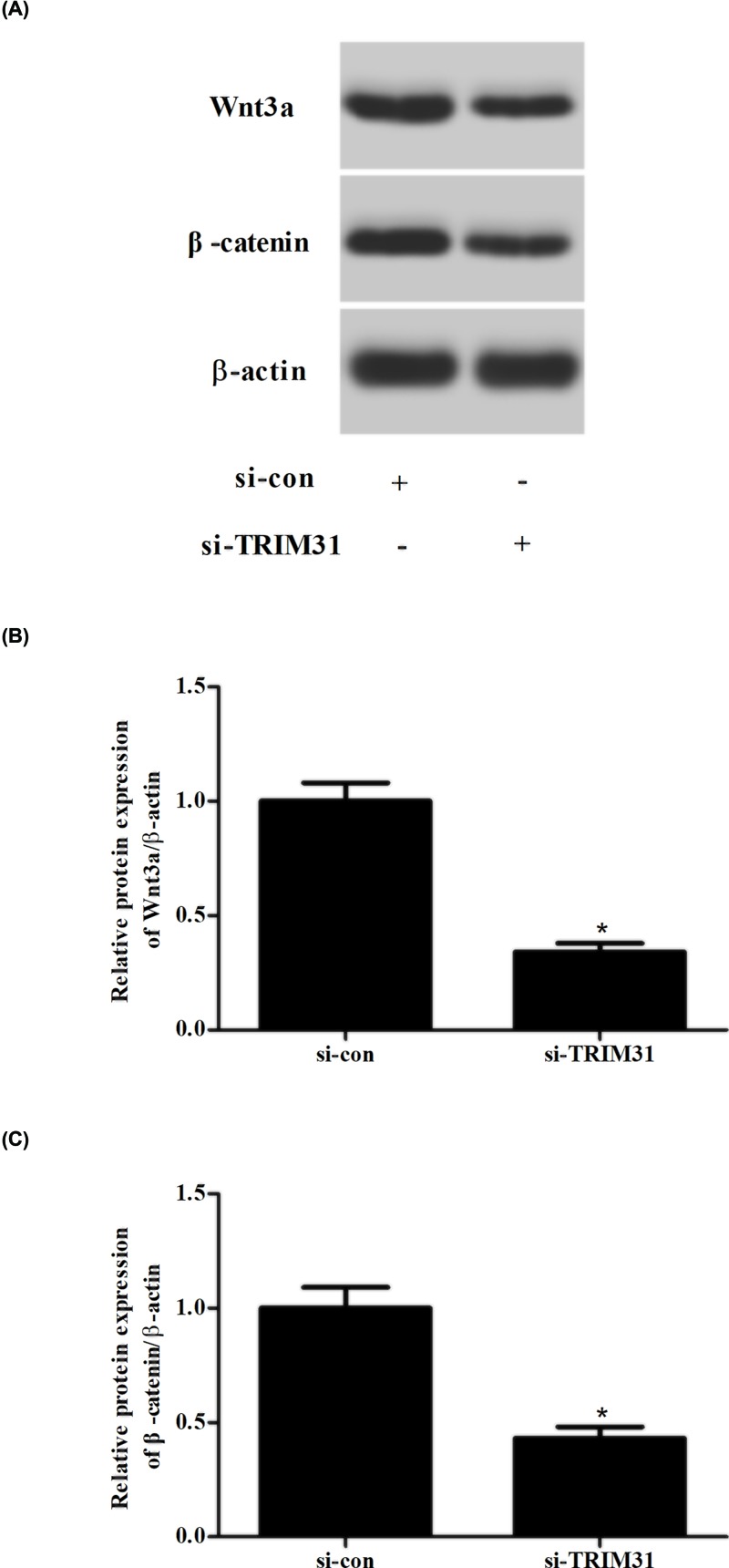
Effects of TRIM31 knockdown on the Wnt/β-catenin signaling pathway in U937 cells (**A**) After transfection with si-TRIM31 or si-con, the expression levels of Wnt3a and β-catenin in U937 cells were measured using Western blot. (**B,C**) Quantification analysis of Wnt3a and β-catenin. *P<0.05 compared with si-con transfected U937 cells.

To further investigate the mechanism of TRIM31, the expression levels of Wnt3a and β-catenin in U937 cells were measured using Western blot. As shown in Figure 5, knockdown of TRIM31 in U937 cells resulted in obvious decrease in Wnt3a and β-catenin expressions. Furthermore, TRIM31 knockdown also significantly down-regulated the protein expression levels of cyclin D1 and c-Myc (Supplementary Figure S3). These data indicating that TRIM31 knockdown inhibited the activation of Wnt/β-catenin signaling pathway in U937 cells.

### LiCl, the Wnt/β-catenin pathway activator, reversed the effects of TRIM31 knockdown on AML cells

**Figure 6:**
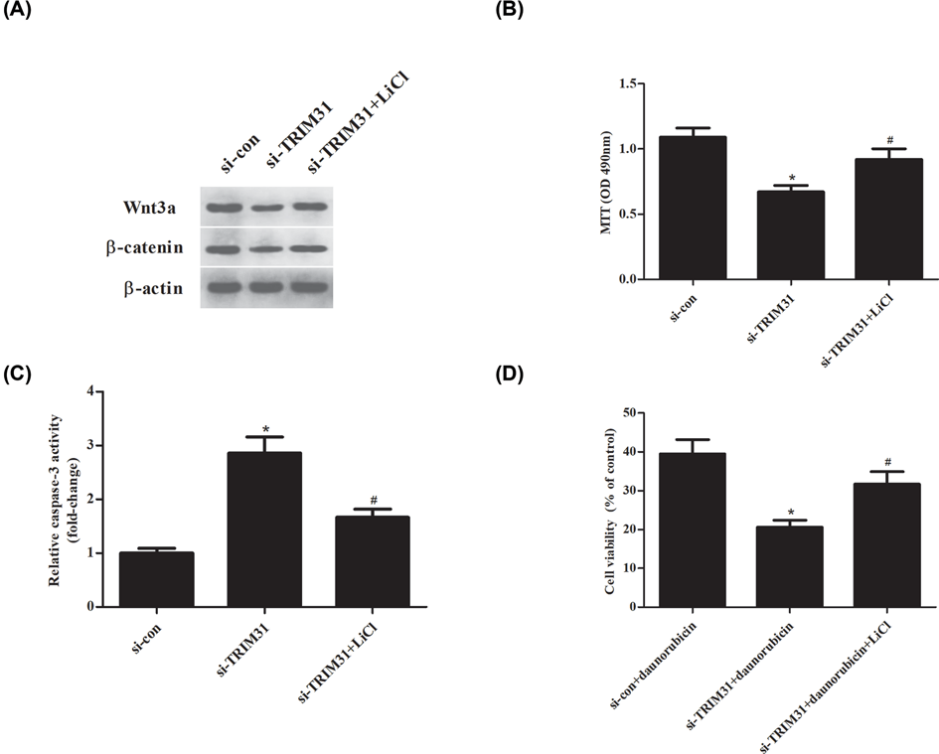
Effects of LiCl on si-TRIM31 transfected U937 cells U937 cells were treated with LiCl to activate Wnt/β-catenin signaling pathway. (**A**) The expression levels of Wnt3a and β-catenin in U937 cells were measured using Western blot. (**B**) MTT assay was performed to detect U937 cells proliferation. (**C**) Flow cytometry was carried out to analyze apoptotic rates of U937 cells. (**D**) Cell viability in U937 cells with or without LiCl treatment. *P<0.05 compared with si-con transfected U937 cells; ^#^*P*<0.05 compared with si-TRIM31 transfected U937 cells.

To further confirm the role of Wnt/β-catenin signaling pathway, U937 cells were treated with LiCl, which is an activator of Wnt/β-catenin signaling pathway. We found that LiCl increased the protein expression levels of Wnt3a and β-catenin in si-TRIM31-transfected U937 cells (Figure 6A). Treatment with LiCl abolished the effects of si-TRIM31 on cell proliferation and caspase-3 activity in U937 cells (Figure 6B,C). As shown in Figure 6D, U937 cells treated with LiCl exhibited higher cell viability to daunorubicin, as compared with the U937 cells transfected with si-TRIM31 to daunorubicin. These findings suggested that activation of Wnt/β-catenin signaling pathway reversed the effects of si-TRIM31 on cell proliferation, apoptosis and daunorubicin sensitivity in U937 cells.

## Discussion

As described in the present study, TRIM31 protein could promote cell proliferation and induce cell apoptosis, as well regulate chemoresistance of AML cells. In addition, the roles of TRIM31 were mediated by the Wnt/β-catenin signaling pathway.

TRIM family is a group of proteins characterized by three novel functional domains: a ‘really interesting new gene’ (RING) domain in the N-terminus, 1 or 2 zinc-finger domains called B boxes (B1 box and B2 box) and a coiled-coil motif [11,12]. Due to the specific nature of TRIM proteins, they have been found to be implicated in a variety of biological processes, such as cell proliferation, cell division and developmental processes and cell metabolism [13]. A growing body of evidence shows that several TRIM members are involved in the tumorigenesis and serve as therapeutic targets for the treatment of cancers [14]. Additionally, several TRIM proteins have been found to be involved in blood cancers, including acute promyelocytic leukemia (APL), B-cell acute lymphocytic leukemia (B-ALL), chronic lymphocytic leukemia (CLL), chronic myeloid leukemia (CML), chronic myelomonocytic leukemia (CMML) and AML [15–18].

Overexpression of TRIM32 in the human promyelogenous leukemic cell line HL60 suppresses cellular proliferation and induces granulocytic differentiation, implying that TRIM32 may be a potentially therapeutic target for APL [16]. TRIM62 levels are significantly lower in AML cells than those in normal CD34-positive cells. Lower TRIM62 level is associated with shorter complete remission duration, shorter event-free and overall survival rates in AML patients, indicating that TRIM62 is an independent adverse prognostic factor in AML [19]. TRIM31 is a cancer-related TRIM protein that has been observed to exert oncogenic activity in several types of cancers [20–22]. Given its involvement in these cancers, we speculated that TRIM31 might represent therapeutic target in AML. To that end, we examined the expression levels of TRIM31 in the blood samples from 34 patients with AML and 34 healthy volunteers. The results showed that TRIM31 was highly expressed in AML patients. Moreover, loss/gain-of-function assays proved that TRIM31 promoted cell proliferation and induced caspase-3 activity AML cells. Knockdown of TRIM31 in AML cells elevated the drug sensitivity to daunorubicin. These findings were in consonance with the notion of TRIM62 as an oncogene in AML.

Transduced Wnt signals are highly conserved and regulate various developmental processes [23,24]. β-catenin is a core downstream protein in the the canonical Wnt pathway. Activation of Wnt leads to β-catenin accumulation and translocation to the nucleus. Here it interacts with T-cell factor/lymphoid enhancer factor (TCF/LEF) transcription factors and thereby regulate the expression of target genes, which are important for embryonic development and proliferation [25,26]. Recently, Wnt/β-catenin pathway has been found to play an important role in the development of AML [27–29]. The β-catenin protein is readily detected in primary AML samples, and is required for the development of leukemia stem cells (LSCs) in AML [30]. Targeting the Wnt/β-catenin pathway may represent a new therapeutic opportunity for the treatment of AML [31]. We found that knockdown of TRIM31 suppressed the activation of Wnt/β-catenin pathway in AML cells. Activation of Wnt/β-catenin signaling pathway by LiCl reversed the effects of si-TRIM31 on AML cells. The results indicated that the oncogenic role of TRIM31 in AML was mediated by the Wnt/β-catenin signaling pathway.

In conclusion, our findings demonstrated that TRIM31 was involved in leukemogenesis and chemoresistance. Wnt/β-catenin signaling pathway played a critical role in the oncogenic potential of TRIM31 in AML. Collectively, TRIM31 might serve as a therapeutic target for the AML treatment.

## Supplementary Material

Supplementary Figures S1-S3

## Abbreviations

AML: acute myeloid leukemia
APL: acute promyelocytic leukemia
HCC: hepatocellular carcinoma
PI: Propidium Iodide
si-TRIM31: TRIM31 siRNA
TRIM: tripartite motif

## References

[1] Kantarjian H. (2016) Acute myeloid leukemia- major progress over four decades, and glimpses into the future. Am. J. Hematol. 91, 131–145 10.1002/ajh.24246 26598393

[2] Jabbour E.J., Estey E. and Kantarjian H.M. (2006) Adult acute myeloid leukemia. Mayo Clin. Proc. Mayo Clin. 81, 247–260 10.4065/81.2.247 16471082

[3] Puumala S.E., Ross J.A., Aplenc R. et al. (2013) Epidemiology of childhood acute myeloid leukemia. Pediatr. Blood Cancer 60, 728–733 10.1002/pbc.24464 23303597 PMC3664189

[4] Pieters R. and Carroll W.L. (2010) Biology and treatment of acute lymphoblastic leukemia. J. Pediatrics 24, 1–18 10.1016/j.hoc.2009.11.014 20113893

[5] Acheampong D.O., Adokoh C.K., Asante D.B. et al. (2018) Immunotherapy for acute myeloid leukemia (AML): a potent alternative therapy. Biomed. Pharmacother. 97, 225–232 10.1016/j.biopha.2017.10.100 29091870

[6] Fabio T. and Maurizio A. (2015) Treatment of pediatric acute lymphoblastic leukemia. Haematologica 62, 61–73 10.1016/j.pcl.2014.09.006 PMC4366417 25435112

[7] Shi G., Lv C., Yang Z. et al. (2019) TRIM31 promotes proliferation, invasion and migration of glioma cells through Akt signaling pathway. Neoplasma 66, 727–735 10.4149/neo_2019_190106N21 31129970

[8] Guo P., Ma X., Zhao W. et al. (2018) TRIM31 is upregulated in hepatocellular carcinoma and promotes disease progression by inducing ubiquitination of TSC1-TSC2 complex. Oncogene 37, 478–488 10.1038/onc.2017.349 28967907

[9] Zhang H., Deng Y., Liang L. et al. (2019) Knockdown Of TRIM31 enhances colorectal cancer radiosensitivity by inducing DNA damage and activating apoptosis. Onco Targets Ther. 12, 8179–8188 10.2147/OTT.S215769 31632068 PMC6781640

[10] Yu C., Chen S., Guo Y. et al. (2018) Oncogenic TRIM31 confers gemcitabine resistance in pancreatic cancer via activating the NF-kappaB signaling pathway. Theranostics 8, 3224–3236 10.7150/thno.23259 29930725 PMC6010981

[11] Nisole S., Stoye J.P. and Saib A. (2005) TRIM family proteins: retroviral restriction and antiviral defence. Nat. Rev. Microbiol. 3, 799–808 10.1038/nrmicro1248 16175175

[12] Reymond A., Meroni G., Fantozzi A. et al. (2001) The tripartite motif family identifies cell compartments. EMBO J. 20, 2140–2151 10.1093/emboj/20.9.2140 11331580 PMC125245

[13] Venuto S. and Merla G. (2019) E3 ubiquitin ligase TRIM proteins, cell cycle and mitosis. Cells 8, pii:E510 10.3390/cells8050510 31137886 PMC6562728

[14] Jaworska A.M., Wlodarczyk N.A., Mackiewicz A. et al. (2020) The role of TRIM family proteins in the regulation of cancer stem cell self-renewal. Stem Cells 38, 165–173 10.1002/stem.3109 31664748 PMC7027504

[15] Crawford L.J., Johnston C.K. and Irvine A.E. (2018) TRIM proteins in blood cancers. J. Cell Commun. Signal. 12, 21–29 10.1007/s12079-017-0423-5 29110249 PMC5842186

[16] Sato T., Okumura F., Iguchi A. et al. (2012) TRIM32 promotes retinoic acid receptor alpha-mediated differentiation in human promyelogenous leukemic cell line HL60. Biochem. Biophys. Res. Commun. 417, 594–600 10.1016/j.bbrc.2011.12.012 22182411

[17] Li L., Qi Y., Ma X. et al. (2018) TRIM22 knockdown suppresses chronic myeloid leukemia via inhibiting PI3K/Akt/mTOR signaling pathway. Cell Biol. Int. 42, 1192–1199 10.1002/cbin.10989 29762880

[18] Chrétien M.L., Legouge C., Martin R.Z. et al. (2016) Trim33/Tif1γ is involved in late stages of granulomonopoiesis in mice. Exp. Hematol. 44, 727–739.e726 27130375 10.1016/j.exphem.2016.04.009

[19] Quintas-Cardama A., Zhang N., Qiu Y.H. et al. (2015) Loss of TRIM62 expression is an independent adverse prognostic factor in acute myeloid leukemia. Clin. Lymphoma Myeloma Leuk. 15, 115–127.e115 10.1016/j.clml.2014.07.011 25248926 PMC4560255

[20] Wang H., Yao L., Gong Y. et al. (2018) TRIM31 regulates chronic inflammation via NF-kappaB signal pathway to promote invasion and metastasis in colorectal cancer. Am. J. Transl. Res. 10, 1247–1259 29736218 PMC5934584

[21] Li H., Zhang Y., Hai J. et al. (2018) Knockdown of TRIM31 suppresses proliferation and invasion of gallbladder cancer cells by down-regulating MMP2/9 through the PI3K/Akt signaling pathway. Biomed. Pharmacother. 103, 1272–1278 10.1016/j.biopha.2018.04.120 29864908

[22] Zhou L., Deng Z.Z., Li H.Y. et al. (2019) TRIM31 promotes glioma proliferation and invasion through activating NF-kappaB pathway. Onco Targets Ther. 12, 2289–2297 10.2147/OTT.S183625 30988633 PMC6441556

[23] Gammons M. and Bienz M. (2018) Multiprotein complexes governing Wnt signal transduction. Curr. Opin. Cell Biol. 51, 42–49 10.1016/j.ceb.2017.10.008 29153704

[24] Zhan T., Rindtorff N. and Boutros M. (2017) Wnt signaling in cancer. Oncogene 36, 1461–1473 10.1038/onc.2016.304 27617575 PMC5357762

[25] Nusse R. and Clevers H. (2017) Wnt/β-Catenin signaling, disease, and emerging therapeutic modalities. Cell 169, 985–999 10.1016/j.cell.2017.05.016 28575679

[26] Chen N. and Wang J. (2018) Wnt/beta-catenin signaling and obesity. Front. Physiol. 9, 792 10.3389/fphys.2018.00792 30065654 PMC6056730

[27] Simon M., Grandage V.L., Linch D.C. et al. (2005) Constitutive activation of the Wnt/beta-catenin signalling pathway in acute myeloid leukaemia. Oncogene 24, 2410–2420 10.1038/sj.onc.1208431 15735743

[28] Kuhn K., Cott C., Bohler S. et al. (2015) The interplay of autophagy and beta-Catenin signaling regulates differentiation in acute myeloid leukemia. Cell Death Discov. 1, 15031–15057 10.1038/cddiscovery.2015.31 27551462 PMC4979480

[29] Ysebaert L., Chicanne G., Demur C. et al. (2006) Expression of beta-catenin by acute myeloid leukemia cells predicts enhanced clonogenic capacities and poor prognosis. Leukemia 20, 1211–1216 10.1038/sj.leu.2404239 16688229

[30] Yingzi W., Krivtsov A.V., Sinha A.U. et al. (2010) The Wnt/beta-catenin pathway is required for the development of leukemia stem cells in AML. Science 327, 1650–1653 20339075 10.1126/science.1186624 PMC3084586

[31] Wang L., You L.-S., Ni W.-M. et al. (2013) β-Catenin and AKT are promising targets for combination therapy in acute myeloid leukemia. Leukemia Res. 37, 1329–1340 10.1016/j.leukres.2013.06.023 23867056

