# Supplementary material for: TRIM31 promotes acute myeloid leukemia progression and sensitivity to daunorubicin through the Wnt/β-catenin signaling
Source: Biosci Rep. 2020 Apr 15;40(4):BSR20194334. doi: 10.1042/BSR20194334 (PMC7160243; doi:10.1042/BSR20194334)
Supplement: Supplementary Figures S1-S3 [file BSR-2019-4334_supp.pdf]

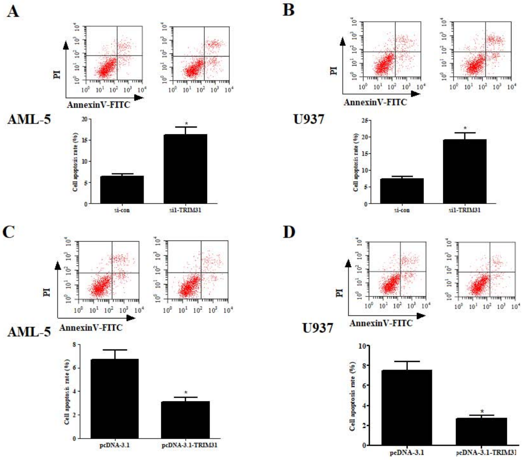

A
B
C
D
PI
AnnexinV-FITC
AML-5
U937
Cell apoptosis rate (%)
si-con
si1-TRIM31
pcDNA-3.1
pcDNA-3.1-TRIM31

Supplementary Figure 1. TRIM31 regulates the apoptosis of AML cells. (A and B) The flow cytometry was carried out to determine cell apoptosis of AML-5 and U937 cells after transfection with si-con or si1-TRIM31. (C and D) The flow cytometry was carried out to determine cell apoptosis of AML-5 and U937 cells after transfection with pcDNA-3.1-TRIM31 or pcDNA-3.1. *p<0.05.

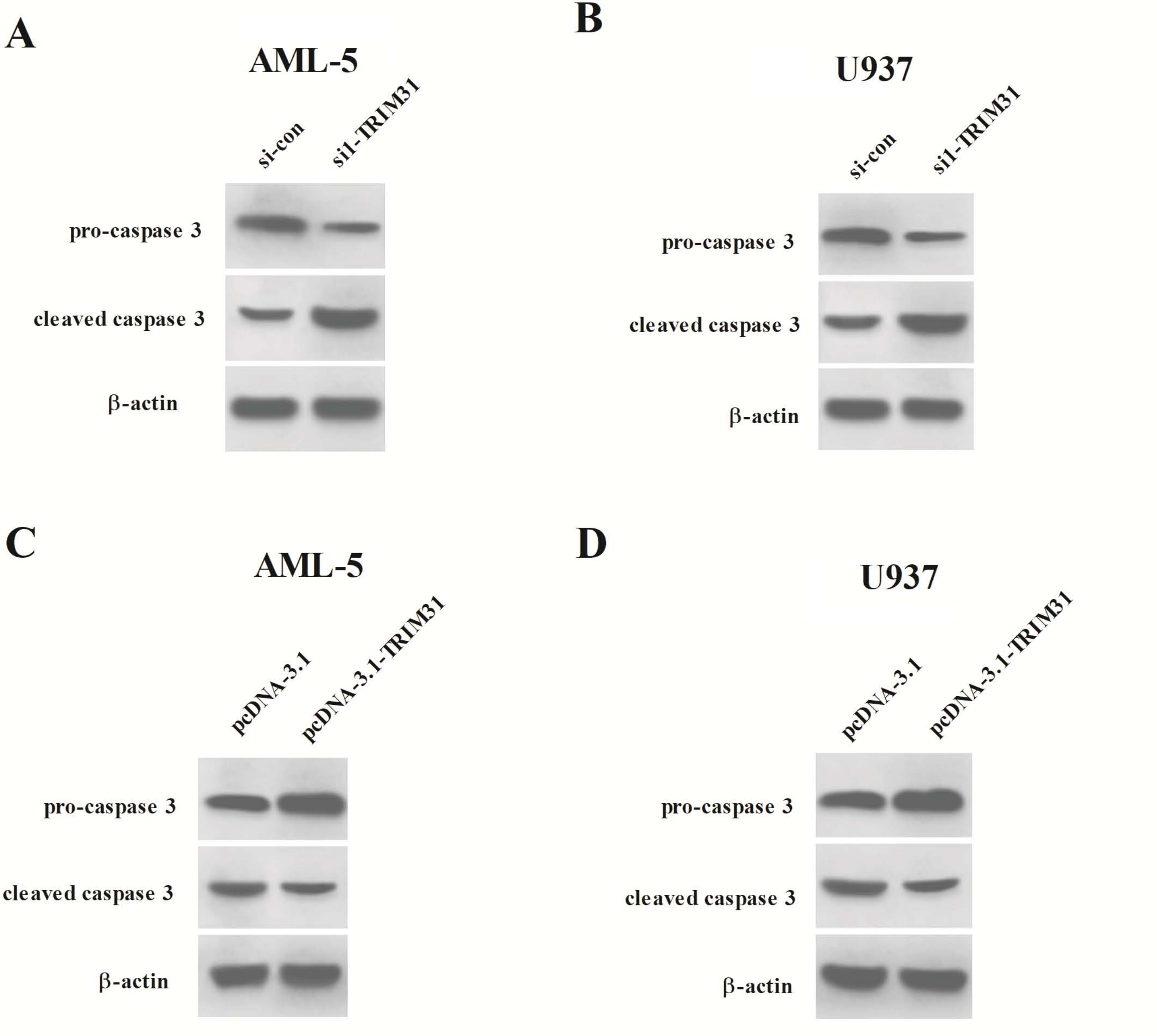
A
AML-5
si-con
si1-TRIM31
pro-caspase 3
cleaved caspase 3
β-actin
B
U937
si-con
si1-TRIM31
pro-caspase 3
cleaved caspase 3
β-actin
C
AML-5
pcDNA-3.1
pcDNA-3.1-TRIM31
pro-caspase 3
cleaved caspase 3
β-actin
D
U937
pcDNA-3.1
pcDNA-3.1-TRIM31
pro-caspase 3
cleaved caspase 3
β-actin

Supplementary Figure 2. TRIM31 regulates the expression of pro-caspase 3 and cleaved caspase 3 in AML cells. (A and B) The expression levels of pro-caspase 3 and cleaved caspase 3 were detected using western blot in AML-5 and U937 cells after transfection with si-con or si1-TRIM31. (C and D) The expression levels of pro-caspase 3 and cleaved caspase 3 were detected using western blot in AML-5 and U937 cells after transfection with pcDNA-3.1-TRIM31 or pcDNA-3.1.

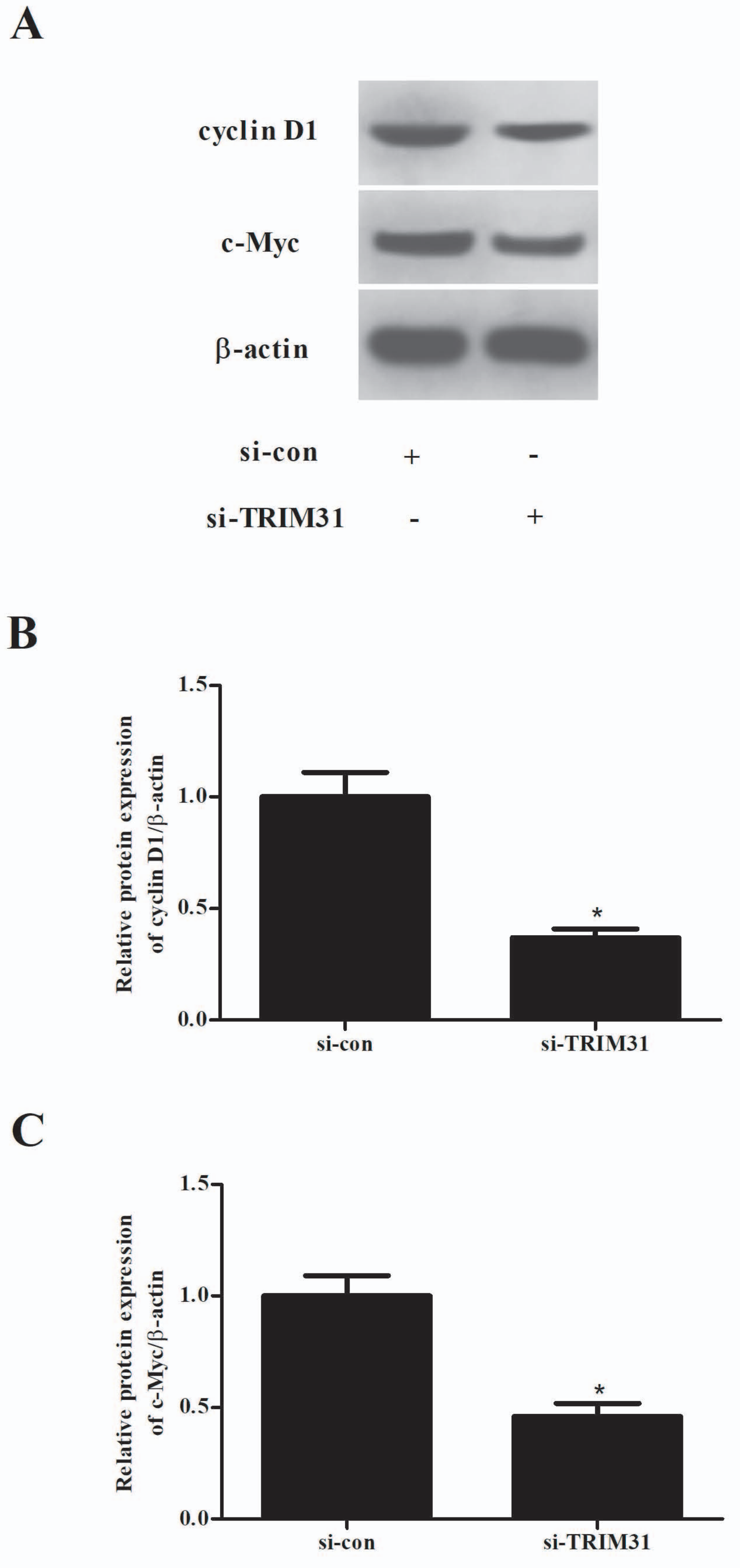
A
cyclin D1
c-Myc
β-actin
si-con + -
si-TRIM31 - +
B
Relative protein expression of cyclin D1/β-actin
1.5
1.0
0.5
0.0
*
si-con
si-TRIM31
C
Relative protein expression of c-Myc/β-actin
1.5
1.0
0.5
0.0
*
si-con
si-TRIM31

Supplementary Figure 3. Effect of TRIM31 knockdown on the downstream targets of Wnt/β-catenin pathway in U937 cells. (A) After transfection with si-TRIM31 or si-con, the expression levels of cyclin D1 and c-Myc were measured using western blot. (B and C) Quantification analysis of cyclin D1 and c-Myc. *$p<0.05$.
